# Neural Response to Theta‐Burst Stimulation Predicts Long‐Term Relapse in Patients With Alcohol Use Disorder: A Pilot fMRI Study

**DOI:** 10.1111/adb.70109

**Published:** 2026-01-11

**Authors:** Jing‐Nan Zhao, Chu‐Yue Zhao, Ying‐Ying Li, Li‐Ping Liu, Zhi‐Jun Liu

**Affiliations:** ^1^ The First Specialized Hospital of Harbin City Harbin China; ^2^ Harbin Center for Disease Control and Prevention Harbin China; ^3^ Queensland Brain Institute The University of Queensland Brisbane QLD Australia

**Keywords:** alcohol use disorder, continuous theta‐burst stimulation, craving, dorsolateral prefrontal cortex, fMRI

## Abstract

Alcohol use disorder (AUD) is characterized by high relapse rates, and relapse is often driven by cue‐induced cravings linked to prefrontal–subcortical network dysregulation. This study investigated the neurobiological effects of inhibitory continuous theta‐burst stimulation (cTBS) targeting the right dorsolateral prefrontal cortex (rDLPFC) in patients with AUD. In a randomized, double‐blind, sham‐controlled trial, 28 patients (16 in the active cTBS group and 12 patients in the sham group) underwent 10 sessions of rDLPFC‐cTBS. fMRI was performed before and after intervention to assess neural responses to alcohol cues, and relapse was monitored for 1 year. The active cTBS group exhibited a significantly lower relapse risk over the 12‐month follow‐up compared to the sham group (HR = 0.210, 95% CI [0.070, 0.633]). A significant group‐by‐intervention interaction was found in the right superior frontal gyrus (*p* = 0.047); active cTBS prevented the cue‐induced hyperactivity that was observed in the sham group, suggesting a network stabilization effect. Furthermore, a machine learning model that was trained on intervention‐induced changes in brain‐wide neural activity accurately predicted long‐term relapse (accuracy: 78.7%; AUC: 0.903). Increased postintervention reactivity to cues in the left medial prefrontal cortex was the strongest predictor of relapse. These findings demonstrate that rDLPFC‐cTBS modulates craving‐related circuits and that the dynamic neural response to treatment is a powerful biomarker for predicting relapse; the findings pave the way for the development of personalized addiction medicine.

## Graphical abstract

This study investigates the effects of active versus sham intervention on a specific measure (Beta value) before and after the treatment. A significant difference was found between the two groups, with a statistical result of *F* = 4.347 and *p* = 0.047. The findings indicate a measurable change from the pre‐test to the post‐test condition following the active intervention.

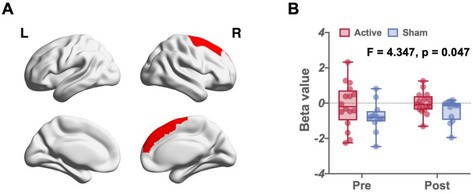

## 1 Introduction

Alcohol use disorder (AUD) is a significant global public health crisis, and it is characterized by chronic relapses that result in substantial morbidity and mortality [1]. Despite the availability of pharmacological and psychosocial interventions, long‐term treatment efficacy is severely hindered by high relapse rates, with a majority of individuals resuming harmful alcohol consumption patterns within months of treatment completion [2, 3]. At the heart of this clinical challenge lies craving, which is an intense and intrusive desire for alcohol that is not only a core diagnostic feature of AUD but also one of the most robust predictors of relapse [4]. The persistently high relapse rate among AUD patients highlights the urgent need for interventions that can precisely modulate the neural circuits underlying this core pathological process.

A prevailing neurobiological model posits that AUD arises not from a focal lesion but from a large‐scale network imbalance, which is primarily characterized by dysregulation between hypoactive prefrontal cognitive control systems and hyperactive subcortical reward and motivation systems [5]. This imbalance fosters a state in which reward‐driven impulses are amplified while the capacity for top‐down inhibition is diminished, culminating in compulsive alcohol‐seeking behaviour [6]. The right dorsolateral prefrontal cortex (rDLPFC) has emerged as a critical node within the brain's executive control network, and it plays pivotal roles in impulse inhibition, goal‐directed behaviour and decision‐making [7]. Many neuroimaging studies have demonstrated structural and functional impairments in the rDLPFC of individuals with AUD. Importantly, causal evidence from neuromodulation studies involving healthy volunteers has shown that transiently disrupting rDLPFC function impairs inhibitory control and directly increases alcohol consumption [8], establishing a direct role of the rDLPFC in regulating alcohol consumption behaviour and confirming that it is a promising therapeutic target.

Repetitive transcranial magnetic stimulation (rTMS) is a powerful tool for directly and focally modulating the activity of cortical networks that are implicated in psychiatric disorders [9]. Continuous theta‐burst stimulation (cTBS) is a particularly efficient rTMS paradigm that can induce a lasting, long‐term depression (LTD)‐like form of neuroplasticity in a very short period, making it uniquely suited for downregulating maladaptive neural activity [10]. The goal is to disrupt the rigid, maladaptive synaptic patterns that maintain the dysfunctional state, thereby creating an opportunity for the brain's homeostatic mechanisms to regain neuroadaptability. However, compared with the conventional rTMS intervention model, the therapeutic efficacy of cTBS still needs to be further evaluated.

To address this gap, we conducted a single‐centre, randomized, double‐blind, sham‐controlled clinical trial to systematically investigate the causal effects of rDLPFC‐cTBS on the neural underpinnings of cue‐induced craving in patients with AUD. We hypothesized that a course of active cTBS, compared with sham stimulation, would significantly reduce subjective alcohol craving. On a neurobiological level, we predicted that this clinical improvement would be mediated by a remodelling of craving‐related neural circuits during an fMRI‐based alcohol cue reactivity task. Specifically, we anticipated that active cTBS would normalize activity within the stimulated rDLPFC. Furthermore, we sought to explore the associations between these cTBS‐induced changes in neural activity and improvements in clinical behaviour, with the ultimate goal of assessing the value of these changes for predicting long‐term relapse outcomes. By linking target engagement at the neural level to clinical efficacy, we aimed to provide robust evidence for a novel therapeutic pathway and establish a potential biomarker for future personalized treatments for patients with AUD.

## 2 Methods

### 2.1 Participants

This pilot study was registered with ChiCTR (identifier: ChiCTR2200065453). Participants were recruited from the alcohol detoxification clinic of the First Specialized Hospital of Harbin. All the participants provided written informed consent, the forms for which were approved by the Institutional Review Board of the First Specialized Hospital of Harbin. The enrolled patients were randomly assigned to receive 10 sessions of active rDLPFC‐cTBS or sham placebo. Patients completed the DSM‐5 AUD Clinical Interview [11] and Alcohol Use Disorders Identification Test (AUDIT) [12] at baseline to assess demographics, medical history, and other substance use. Patients were contacted by telephone at 1, 3 (90 days), 6 (180 days), 9 (270 days), and 12 (360 days) months after treatment completion to assess relapse. Finally, this study included 16 patients in the active group and 12 in the sham group. The demographic information of the patients is shown in Table 1.

**TABLE 1:** Demographic variables in this study.

		Active	Sham	*t/* ${Χ}^{2}$	*p*
		M (SD)	M (SD)		
Gender (%)	F	2 (12.5)	2 (16.7)	0.097	0.755
	M	14 (87.5)	10 (83.3)	/	/
Age		46.50 (8.65)	48.58 (10.68)	−0.571	0.573
Weight		69.44 (15.20)	62.08 (7.82)	1.527	0.139
Years of education		10.27 (2.09)	10.33 (1.97)	−0.085	0.933
AUDIT		33.13 (6.21)	25.17 (10.88)	2.397	0.024
CLWA		26.13 (13.71)	21.08 (11.70)	1.013	0.321
FTND		3.07 (3.24)	4.50 (3.09)	−1.166	0.255
PSS‐10		29.53 (9.46)	29.00 (10.23)	0.140	0.889
Subjective sleep quality		2.60 (1.06)	1.92 (1.31)	1.502	0.146
Sleep latency		2.20 (1.15)	2.17 (1.03)	0.078	0.938
Sleep persistence		1.33 (1.29)	1.33 (1.16)	< 0.001	1.000
Sleep efficiency		1.60 (1.40)	0.83 (1.19)	1.505	0.145
Sleep turbulence		1.33 (0.62)	1.08 (0.52)	1.124	0.272
PSQI		12.13 (4.85)	9.42 (3.26)	1.659	0.110
Habit		5.44 (1.47)	4.46 (2.10)	1.433	0.164
Reward		5.69 (0.81)	5.01 (1.29)	1.661	0.109
Fear		4.06 (0.93)	3.71 (0.92)	0.972	0.340

*Note:* Data are presented as the means (standard deviations) for continuous variables and frequencies (percentages) for categorical variables (e.g., gender). The ‘Test Statistic’ column reports *t*‐values for continuous variables (independent t tests) and *χ* ^2^ values for categorical variables (chi‐square tests).

Abbreviations: AUDIT = Alcohol Use Disorders Identification Test; CLWA = Craving for Liquor Withdrawal Assessment; FTND = Fagerström Test for Nicotine Dependence; PSS‐10 = Perceived Stress Scale‐10; PSQI = Pittsburgh Sleep Quality Index. Statistical significance was set to *p* < 0.05.

### 2.2 Inclusion/Exclusion Criteria

The primary inclusion criteria were age between 18 and 60 years, primary diagnosis of AUD according to DSM‐5 criteria, at least a junior high school education, and the ability to provide written informed consent. The exclusion criteria were as follows: (1) current acute alcohol withdrawal or severe withdrawal symptoms; (2) a history of epilepsy or seizure disorders; (3) any contraindications for transcranial magnetic stimulation (TMS) or magnetic resonance imaging (MRI), such as the presence of metallic implants; and (4) a current severe psychiatric illness or a history of intellectual disability.

### 2.3 cTBS Session Execution

A total of 10 intervention sessions were administered over two consecutive weeks (one session per day, Monday to Friday). All the procedures were conducted using a certified magnetic stimulator equipped with a fig. 8 cooling coil (Wuhan Yiruide Medical Co. Ltd., Wuhan, China). To ensure individualized and precise treatment, each participant's resting motor threshold (RMT) was determined prior to the first session. The RMT was defined as the minimum stimulator output required to induce a motor‐evoked potential (MEP) of at least 50 μV in five out of 10 consecutive trials from the contralateral abductor pollicis brevis (APB) muscle hotspot.

The stimulation target for both the active and sham groups was the rDLPFC. The coil was positioned using the F4 coordinate of the International 10–20 EEG system as a scalp landmark. For the active intervention, cTBS was delivered at an intensity of 100% of the individual's RMT. The cTBS protocol consisted of three pulse bursts at 50 Hz, repeated at a 5‐Hz theta rhythm, delivering a total of 600 pulses over a 40‐s duration. The sham cTBS intervention was designed to maintain a double‐blind design. The coil was placed at the same F4 location but oriented perpendicularly (90°) to the scalp. This method produces the same device sounds and scalp sensations as active stimulation but does not generate an effective magnetic field that penetrates the skull. The treatment schedule and all other procedural aspects were identical across both groups to control for nonspecific therapeutic effects. After the final session, the participants were asked to rate whether they believed they had received active treatment or sham treatment and how confident they were in their assessment.

### 2.4 Alcohol Cue Reactivity fMRI Task

A visual fMRI alcohol cue reactivity task was employed to measure changes in neural activation in response to alcohol versus non‐alcohol stimuli [13]. The task utilized a block design consisting of four distinct conditions that were presented in a pseudorandomized order: (1) alcohol‐related cues (images of alcoholic beverages and drinking contexts), (2) neutral cues (images of non‐alcoholic beverages and everyday objects matched for visual complexity), (3) mosaicized alcohol cues and (4) a fixation cross baseline. The mosaicized images, which were created by scrambling the alcohol cues, served as a low‐level visual control for basic features such as colour and luminance.

While in the scanner, the participants were instructed to view the images passively. The task consisted of three 30‐s blocks for each of the four conditions. Within each block, images from the corresponding category were continuously presented. The inclusion of both neutral and mosaicized control conditions allowed for the specific isolation of neural activity related to the high‐level cognitive and emotional processing of alcohol cues, which are distinct from general visual processing.

### 2.5 Image Acquisition, Preprocessing and Analysis

Functional images were acquired with a gradient‐echo planar imaging (EPI) sequence on a 3T GE Discovery MR750 system. The acquisition parameters were as follows: repetition/echo time (TR/TE) = 2000/45 ms; flip angle (FA) = 90°; field of view (FOV) = 192 mm; matrix = 64 × 64; and 32 contiguous 4‐mm‐thick slices.

The functional MRI data were preprocessed using FMRIPREP (version 24.1.0) [14]. For each participant, the T1‐weighted structural image was corrected for intensity nonuniformity, skull‐stripped and segmented into grey matter, white matter and cerebrospinal fluid. It was then spatially normalized to the ICBM 152 standard template using nonlinear registration (ANTs). The functional BOLD data underwent head motion correction (FSL mcflirt) and were coregistered to the T1w reference using a boundary‐based registration algorithm. Following normalization to standard space, the functional images were resampled to 2 × 2 × 2‐mm^3^ isotropic voxels and spatially smoothed using a 6.0‐mm FWHM Gaussian kernel. All the transformations were composed and applied in a single interpolation step to preserve the data quality.

For each participant, a first‐level GLM was specified and fitted for each of the two fMRI sessions (pre‐ and postintervention). The model included regressors for each experimental condition (i.e., alcohol cues, mosaicized alcohol cues, neutral cues, and fixations), which were created by convolving the event onset and duration with the canonical hemodynamic response function (HRF) from SPM. To account for motion‐related artefacts, the six head motion parameters that were generated during preprocessing were included as nuisance regressors in the design matrix. From the fitted models, a statistical contrast map (alcohol cues—0.5*mosaicized alcohol cues—0.5*neutral cues) was computed for each participant and each session. These individual contrast maps served as the input for all subsequent second‐level group analyses.

### 2.6 Statistical Analysis

To assess long‐term outcomes and identify predictors for the time to relapse, we implemented a Cox proportional hazards model. Relapse was defined as drinking severity returning to a level similar to or worse than preintervention. The model incorporated group as the primary predictor, along with baseline covariates including sex, age and Perceived Stress Scale‐10 (PSS‐10). Two observations were excluded from the Cox analysis due to loss to follow‐up. For beta map, we used the Harvard–Oxford Brain Atlas to segment it into 112 structural regions. We performed a repeated‐measures ANOVA on the right superior frontal gyrus (rSFG) to assess changes in neural activity associated with the stimulation target after intervention. In addition, we used brain‐wide neural activity as an input and used a transformer‐based probabilistic neural network algorithm to predict patient relapse during follow‐up.

## 3 Results

**FIGURE 1:**
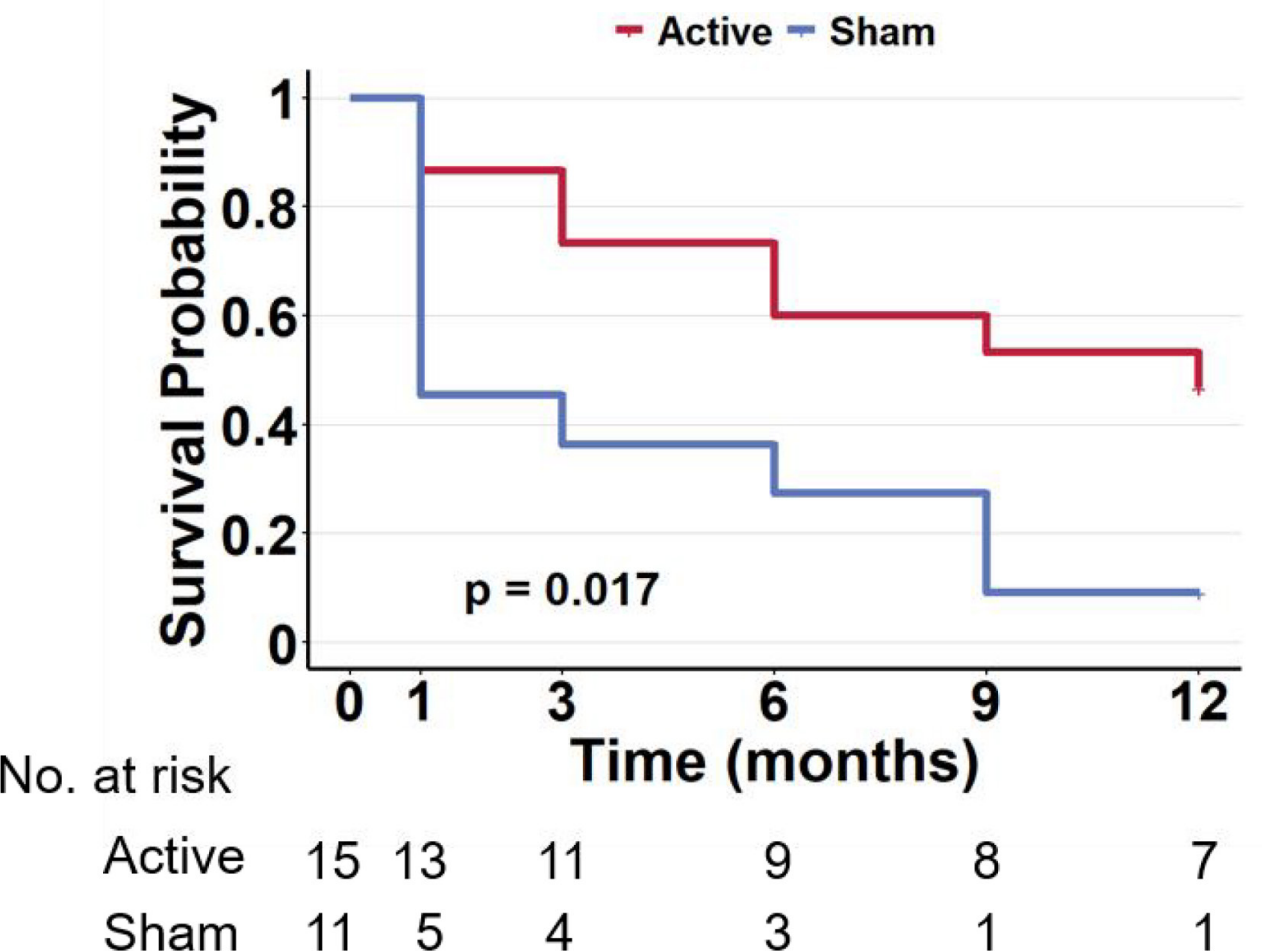
Effects of cTBS on clinical outcomes. Kaplan–Meier survival curves for the 12‐month follow‐up period. The curves depict the cumulative probability of remaining relapse‐free over time for the active cTBS (red line) and sham (blue line) groups. The log‐rank test revealed a significant difference between the two groups, with the active cTBS group showing a lower relapse rate.

Turning to the long‐term follow‐up, the Cox proportional hazards analysis confirmed that the treatment group was a significant factor in predicting the time to relapse. Crucially, participants in the active group demonstrated a significantly reduced likelihood of relapse relative to the sham group (HR = 0.210, 95% CI [0.070, 0.633], *p* = 0.006). This finding was visually supported by Kaplan–Meier survival plots. Furthermore, the overall fit of the model, as assessed by the score (log‐rank) test, approached statistical significance (*p* = 0.017) (Figure 1).

**FIGURE 2:**
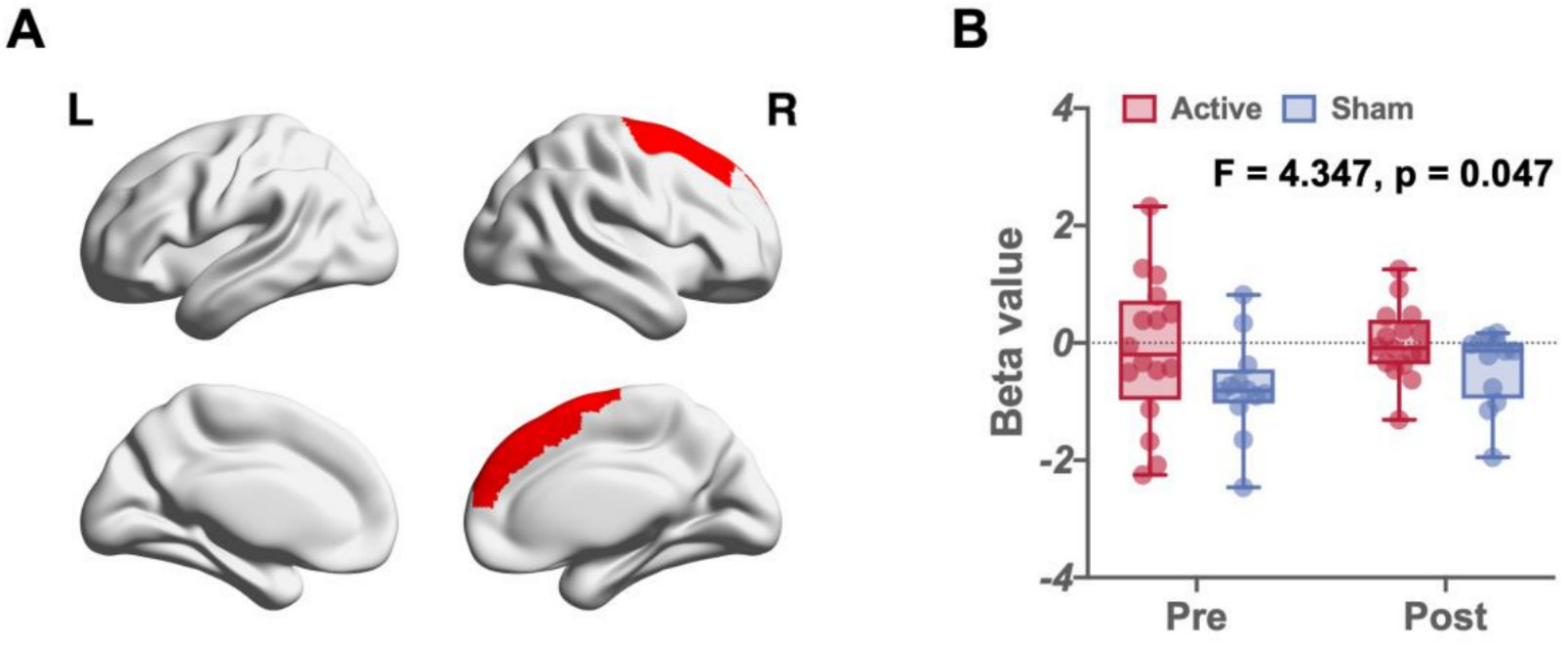
The group–intervention interaction in the right superior frontal gyrus. Panel A shows the spatial location of the right superior anterior gyrus; Panel B shows the mean BOLD signal change in the rSFG, with error bars representing the standard error of the mean.

When participants viewed the craving cue, we detected a group–intervention interaction effect on neural activity in the right superior frontal gyrus (*F* = 4.347, *p* = 0.047). Simple effects analysis revealed that the sham group exhibited increased neural activity in the posttest, whereas this activity was effectively inhibited in the active group (Figure 2).

**FIGURE 3:**
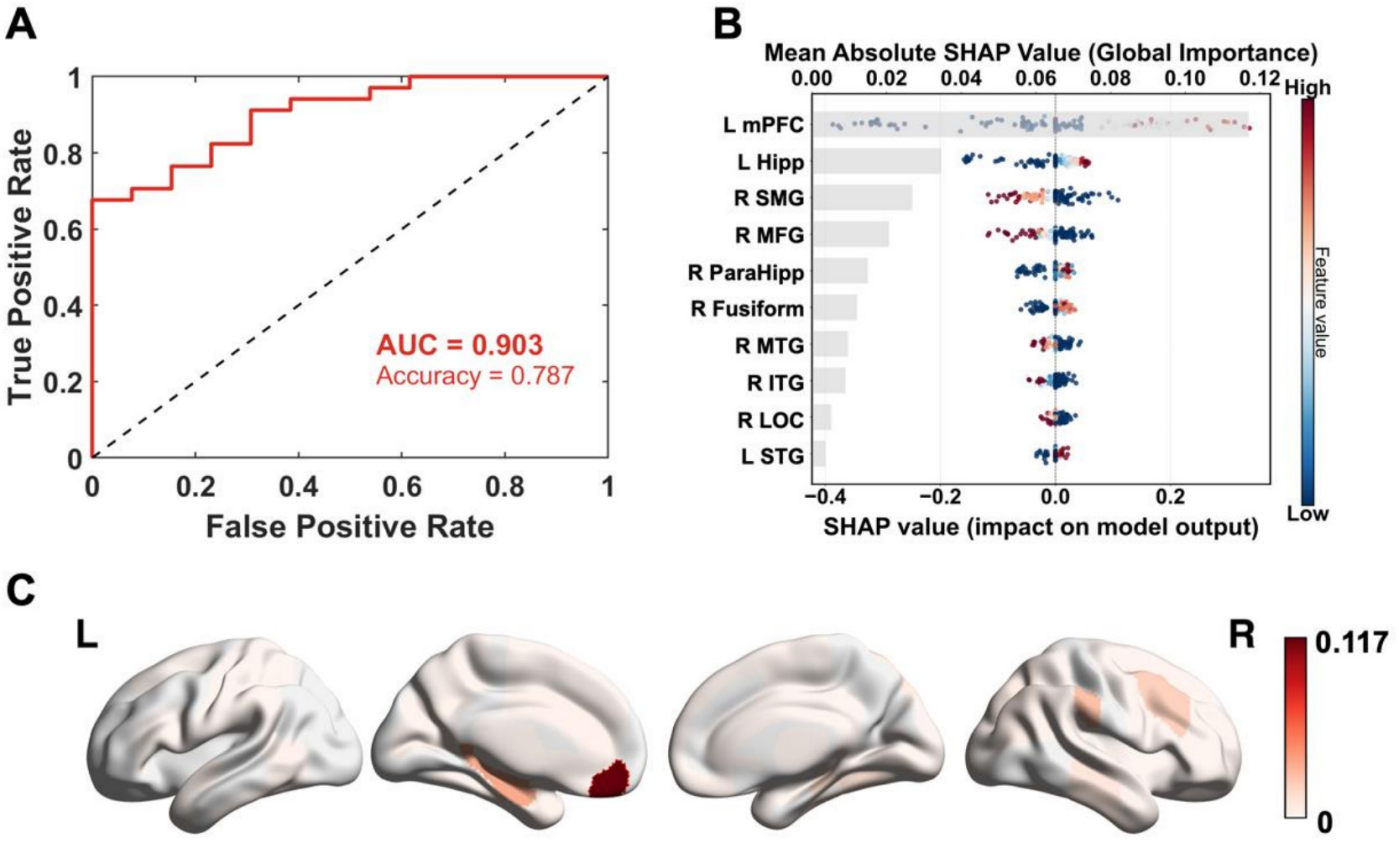
Craving‐related neural activity predicts long‐term relapse in patients. Panel A shows the performance of the prediction model; Panel B shows the SHAP values for the top 10 ROIs, where positive values indicate increased neural activity associated with higher relapse risk; Panel C shows the SHAP value of the whole brain. L Hipp, left hippocampus; L mPFC, left medial frontal cortex; L STG, superior temporal gyrus, anterior division; R ITG, right inferior temporal gyrus, posterior division; R LOC, right lateral occipital cortex, superior division; R MFG, right middle frontal gyrus; R MTG, middle temporal gyrus, posterior division; R ParaHipp, parahippocampal gyrus, posterior division; R SMG, right supramarginal gyrus, anterior division.

We then examined whether changes in craving‐related neural activity could predict long‐term relapse in patients. We found that the changes in craving‐related neural activity before and after the intervention could accurately predict relapse in 78.7% of patients, and the area under the operator characteristic curve (AUC) of the prediction model was 0.903. Figure 3 shows the SHAP summary plot of the top 10 ROIs that contribute most to the model prediction. We found that the greater the difference in neural activity in the left medial frontal cortex (posttest‐baseline) was, the greater the likelihood that the patient would relapse. Figure 3 shows the SHAP average of the whole brain. ROIs with high predicted contributions included the medial frontal cortex (mPFC), hippocampus, and supramarginal gyrus (anterior).

## 4 Discussion

This randomized, sham‐controlled trial provides novel insights into the neurobiological mechanisms by which cTBS affects AUD and establishes a powerful, brain‐based biomarker for predicting long‐term relapse. Our study yields two principal findings. First, we provide causal evidence that an inhibitory cTBS protocol targeting the rDLPFC normalizes cue‐induced hyperactivity within the prefrontal executive control network. Second, we demonstrate that the dynamic changes in the brain‐wide neural response to alcohol cues following the intervention can predict relapse outcomes. This is the first study to mechanistically link rDLPFC‐cTBS to the modulation of craving‐related neural circuits in AUD patients and to develop a prognostic biomarker on the basis of the brain's response to a therapeutic challenge.

A significant group‐by‐intervention interaction was found in the rSFG, which was functionally linked to our rDLPFC target. Although the sham group exhibited a progressive increase in neural activation in response to alcohol cues from baseline to postintervention, the responses in the active cTBS group remained stable. This pattern suggests a more nuanced mechanism than simple inhibition. The increasing reactivity in the sham group likely reflects a natural history of early abstinence, where the brain often becomes sensitized and increasingly hyperreactive to drug‐related stimuli [15, 16]. In this context, the effect of cTBS may be better understood not as direct suppression. By inducing LTD‐like plasticity, cTBS may prevent the maladaptive escalation of cue‐driven excitability, effectively conferring a protective effect that stabilizes the executive control network against pathological sensitization.

Our study leveraged machine learning to move beyond group‐level effects and predict individual patient outcomes. The most powerful predictor of relapse was a postintervention increase in cue reactivity within the left mPFC. This result can be understood as a downstream extension of the cTBS intervention, demonstrating how modulatory effects can propagate from the superficial rDLPFC target to deeper, interconnected prefrontal areas. The predictive direction of this change in mPFC activity is consistent with the activation changes that were observed at the stimulation target, collectively illustrating prefrontal dynamics. Functionally, the mPFC is a central hub for attributing motivational salience to drug cues and generating the compulsive drive to seek them out [17, 18]. This suggests that relapse is driven by a failure to contain the powerful, cue‐driven motivational signals originating from the mPFC, identifying it as a critical locus of relapse vulnerability and a key target for future, potentially more direct, interventions.

Regarding the contralateralization outcome, although the intervention target was the right DLPFC, the strongest predictor of relapse was from the contralateral left mPFC. We propose that this reflects a network‐level modulation rather than a purely local effect, driven by the principles of hemispheric competition and disinhibition. The inhibitory cTBS protocol applied to the rDLPFC may have dampened its normal inhibitory influence on the left hemisphere, which is often mediated via transcallosal pathways [19]. This reduction in cross‐hemispheric inhibition could, in turn, lead to a ‘disinhibition’ of specific nodes in the contralateral hemisphere, such as the left mPFC. The fact that the predictive contributions from the left mPFC and the right MFG pointed in opposite directions seems to support this interpretation of a complex interhemispheric rebalancing.

In addition, we showed that changes in the hippocampus and the right supramarginal gyrus are significant contributors to the risk of relapse, revealing the multifaceted nature of this vulnerability. The hippocampus is crucial for encoding and retrieving the powerful, context‐dependent ‘craving memories’ that link environmental cues to the rewarding effects of alcohol [20, 21]. Furthermore, the supramarginal gyrus has been implicated in higher order metacognitive functions, including self‐other distinction and attentional control as part of the frontoparietal network [22]. Its role in predicting relapse may signify a deficit in cognitive distancing, that is, an inability to perceive one's own craving as a transient, manageable mental event rather than an urgent, egosyntonic command. These lines of evidence may suggest that impaired self‐awareness about internal states is a previously underappreciated cognitive vulnerability for relapse.

Several limitations of this pilot study should be acknowledged. First, the most significant limitation is the relatively small sample size (*n* = 28). This was due in part to resource constraints, but it also reflects the substantial challenges in recruitment and patient retention associated with conducting an intensive 12‐month long‐term follow‐up. A small sample size may limit our statistical power to detect smaller effect sizes and could reduce the generalizability of our findings to broader AUD populations, particularly those with varying severities of AUD or comorbidities. Since this was a single‐centre study, the results should be replicated in more diverse clinical settings. This report focused primarily on the neurobiological predictors of relapse, and a more comprehensive analysis correlating these neural changes with granular clinical outcomes is warranted. Finally, while the prediction model demonstrated a strong predictive relationship, the link between the change in mPFC activity and relapse remained correlational. Future studies should employ larger, multicentre cohorts to validate these findings and explore the integration of cTBS with other interventions, such as cognitive–behavioural therapy, to increase treatment efficacy.

In conclusion, this study demonstrates that rDLPFC‐cTBS effectively modulates cue‐related neural activity and, most importantly, that the resulting change in brain function serves as a highly accurate predictor of long‐term relapse. This work highlights the potential of using neuroimaging not only to understand treatment mechanisms but also as a clinical tool to predict and potentially personalize treatments for addiction.

## Author Contributions

**Jing‐Nan Zhao:** writing – review and editing, writing – original draft and supervisor. **Chu‐Yue Zhao:** methodology and formal analysis. **Ying‐ying Li:** writing review and editing and data curation. **Li‐Ping Liu:** integrated data and formal analysis. **Zhi‐Jun Liu:** writing review and editing.

## Funding

The authors have nothing to report.

## Ethics Statement

This research was approved by the Research Ethics Committee of Harbin First Specialized Hospital (IRB2019–003). All the participant data were anonymized and stored securely in compliance with institutional guidelines. The participants received no financial compensation but were provided with transportation support.

## Conflicts of Interest

The authors declare no conflicts of interest.

## Data Availability

The data that support the findings of this study are available from the corresponding author upon reasonable request.
